# How do first‐line managers in elderly care experience their work situation from a structural and psychological empowerment perspective? An interview study

**DOI:** 10.1111/jonm.12793

**Published:** 2019-06-09

**Authors:** Heidi Hagerman, Maria Engström, Barbro Wadensten, Bernice Skytt

**Affiliations:** ^1^ Faculty of Health and Occupational Studies University of Gävle Gävle Sweden; ^2^ Department of Public Health and Caring Sciences Uppsala University Uppsala Sweden; ^3^ Nursing Department, Medicine and Health College Lishui University Lishui China

**Keywords:** elderly care, empowerment, first‐line manager, structures of proportions, work situation

## Abstract

**Background:**

The work situation for first‐line managers in elderly care is complex and challenging. Little is known about these managers' work situation from a structural and psychological empowerment perspective.

**Aim:**

To describe first‐line managers' experiences of their work situation in elderly care from a structural and psychological empowerment perspective.

**Method:**

Interviews from 14 female first‐line managers were analysed using qualitative content analysis.

**Results:**

The theme described the managers' work situation as “It's not easy, but it's worth it.” In the four subthemes, the managers described their work in terms of “Enjoying a meaningful job,” “A complex and demanding responsibility that allows great authority within set boundaries,” “Supported by other persons, organisational preconditions and confidence in their own abilities” and “Lacking organisational preconditions, but developing strategies for dealing with the situations.”

**Conclusion:**

The managers described having various amounts of access to structural empowerment and experienced a feeling of meaning, competence, self‐determination and impact, that is, psychological empowerment in their work.

**Implications for nursing management:**

It is vital that first‐line managers have access to organisational support. Therefore, upper management and first‐line managers need to engage in continuous dialogue to customize the support given to each first‐line manager.

## 1 BACKGROUND

First‐line managers (FLMs) play a crucial role in elderly care (Dwyer, 2011). They work in publicly funded and politically run organisations, in between upper management/politicians and the staff and clients. FLMs are responsible for their unit's services, daily operations, finances, staff and clients (Hagerman, Engström, Häggström, Wadensten, & Skytt, 2015). Regarding their managerial position, managers have described having significant influence on their subordinates' work conditions and well‐being (Cummings et al., 2018; Holm & Severinsson, 2014) as well as on the quality of care (Skytt, Hagerman, Strömberg, & Engström, 2015) and patient safety (Wong, Cummings, & Ducharme, 2013). Regarding their own situation, one review reported that managers working with older persons and in hospitals have rated moderate stress levels in their work due to high job demands, heavy workloads, financial responsibilities and lack of resources (Labrague, McEnroe‐Petitte, Leocadio, Van Bogaert, & Cummings, 2018). However, a review by Lee and Cummings (2008) reported positive relationships between nurse managers' job satisfaction and empowerment, span of control and organisational support. Another review suggested that managers' working life is influenced by three general categories of factors: organisational factors (e.g., structural empowerment), role‐related factors (e.g., factors inherent to managerial roles) and personal factors (e.g., psychological empowerment; Brown, Fraser, Wong, Muise, & Cummings, 2013).

During recent decades, elderly care has experienced vast organisational changes and it will face new challenges in future. FLMs have a difficult work situation, and the elderly population and their need for care are increasing (Thelin & Wolmesjö, 2014). When studying the working life of managers in different municipal departments, managers in elderly care reported more unhealthy work conditions than managers in other departments did (Berntson, Wallin, & Härenstam, 2012). For example, they reported being pressed by high demands combined with limited resources and low support from upper management and subordinates. They also reported higher stress and lower motivation, work ability and goal achievement (Berntson et al., 2012). High workload and reduced possibilities to impact their work have been reported by managers in elderly care in different countries (Dwyer, 2011; Håkanson et al., 2014; Kristiansen, Westeren, Obstfelder, & Lotherington, 2016). However, although the work situation for FLMs in elderly care is complex, FLMs experienced their work as a positive challenge (Hagerman et al., 2015), and one review found that FLMs were highly motivated to work in elderly care and to produce high‐quality care for older persons (Dwyer, 2011).

### 1.1 Theoretical framework

Kanter's theory (1993) of structural empowerment consists of four components: opportunities (to develop knowledge and skills and grow professionally), information (organisational and workplace knowledge), resources (time, finances, staff and materials) and support (feedback and guidance in decision‐making from colleagues, management and subordinates). Access to these structures is influenced by the individual's formal (work‐role‐related) and informal (personal‐networks‐related) power. Whereas structural empowerment influences individuals' organisational effectiveness and well‐being, psychological empowerment describes their personal response to their work and role in the organisation. According to Spreitzer (1995), psychological empowerment consists of the dimensions: meaning (the fit between the individual's own ideas, values and behaviours and the requirements of the role), competence (the individual's confidence in his/her ability to perform in the role), self‐determination (the individual's sense of autonomy and ability to make his/her own decisions about work) and impact (the ability to influence strategic, administrative or operative outcomes at work). Structural empowerment contributes to psychological empowerment, which in turn can contribute to positive work behaviours and well‐being among nurses (Wagner et al., 2010). Therefore, it is important to have access to empowering structures, as access may increase the organisational effectiveness and well‐being of managers.

The present study is a part of a larger research project focused on FLMs' working life from an empowerment perspective. The longitudinal studies (Hagerman, Skytt, Wadensten, Högberg, & Engström, 2016, Hagerman, Högberg, Skytt, Wadensten, & Engström, 2017) showed that the more access the FLMs had to structural empowerment over time, the more likely they were to feel psychologically empowered, resulting in lower stress symptoms and higher ratings for their own self‐rated leadership‐management performance over time (Hagerman et al., 2016)NN3 Blinded for review). Furthermore, the more access the FLMs had to structural empowerment at time 1, the more access their subordinates had to structural empowerment at time 2 and the higher the subordinates rated their FLM's leadership‐management performance at time 2, controlling for psychological empowerment (Hagerman et al., 2017). In addition, the cross‐sectional study found staff assessment of empowerment to be related to client satisfaction with care (Engström, Högberg, Strömberg, Hagerman, & Skytt, 2019). In the interview studies, the FLMs perceived that staff had varying degrees of access to empowering structures (Skytt et al., 2015), and male FLMs reported they needed better access to resources, support and information (Hagerman et al., 2015). However, most previous empowerment studies have been quantitative, using a hospital setting, and few studies (Hagerman et al., 2015; Kristiansen et al., 2016) have investigated working life among FLMs in elderly care. By interviewing FLMs, a more in‐depth understanding of their experiences of their work situation from an empowerment perspective may emerge. Therefore, a qualitative interview study was conducted, the goal being to describe FLMs' experiences of their work situation in elderly care from a structural and psychological empowerment perspective.

## 2 METHOD

### 2.1 Design and sample

A descriptive design with a qualitative approach was used. As the FLMs' experiences of their work situation in an elderly care context were of interest in the study, qualitative content analysis was selected because it focuses on both the subject and the context (Graneheim & Lundman, 2004). Purposive sampling was carried out, and fourteen FLMs working in elderly care in Sweden participated in the study, Table 1.

**Table 1:** Characteristics of the FLMs and their workplaces

	Range (median)
Age, years	34–65 (49)
Years of FLM experience	2.5–26 (9)
Number of subordinates	16–65 (38)
Educational background, *n*	
Nurse	5
Social worker	7
Economist	1
Physiotherapist	1
Education in leadership, *n*	
In‐service training	10
University‐level training	4
Workplaces, *n*	
Municipal elderly care	12
Private elderly care	2

Abbreviation: *n*: number.

### 2.2 Data collection

Between April–June 2012, semi‐structured interviews were conducted at a place the participants chose: their workplace (*n* = 13) and the municipal hall (*n* = 1). The interviews were audio‐recorded, lasted between 75–140 min and they were based on an interview guide inspired by empowerment theories (Kanter, 1993; Spreitzer, 1995). Open‐ended questions and probes were used to allow participants to speak freely about their work situation. Table 2 presents examples of interview questions.

**Table 2:** Examples of interview questions in the interview guide

Examples of questions	Focusing on
Can you describe what you believe comprises your work as an FLM?	
Can you talk about your resources?	Structural empowerment
Can you talk about the support you get?	Structural empowerment
How do you view your competence in relation to your work?	Psychological empowerment
What aspects of your work do you find meaningful?	Psychological empowerment
Please tell me more	Probe
What does that mean for you?	Probe

### 2.3 Ethics

The Regional Ethical Review Board in Uppsala, Sweden (reg. no. 2010/192), approved the study. All FLMs received oral and written information about the study. They were informed that the study had ethical approval, that participation was strictly voluntary and that they could terminate participation at any time without giving a reason. They were also assured confidentiality. All of the FLMs gave their written consent to participate in the study.

### 2.4 Data analysis

Qualitative content analysis guided by Graneheim and Lundman (2004) was used to analyse the data. The focus of the interview texts was twofold. For the purpose of the present study, the text concerning the female FLMs' experiences of their own empowerment and work situation was extracted and analysed, while FLMs' perceptions regarding their staff's access to structural empowerment have been published (Skytt et al., 2015). The verbatim‐transcribed interviews were read, and meaning units based on the study aim were extracted and labelled with codes. Codes were sorted by similarities and differences into four subthemes, which were abstracted into a theme.

### 2.5 Trustworthiness

To enhance credibility (Graneheim & Lundman, 2004), participants with different kinds of background characteristics were selected. To reduce the potential risk of the researcher's interpretations affecting the data, the whole research group was involved in the analysis. Discussions about the process continued until consensus was reached. During the analysis, the research group went back and forth to the original texts to verify that nothing had changed in meaning or was missing. To enhance dependability, the data collection was performed by the first author within a short time interval to minimize the risk of the data changing over time. To minimize the risk of inconsistency, the informants were encouraged to speak freely about their work during the interviews, and the interview guide was used as a checklist to ensure that all topics were discussed. The method and the result sections have been described in detail to enable the reader to decide whether the results, exemplified using verbatim quotations, are transferable to another group or context (Graneheim & Lundman, 2004).

## 3 RESULTS

### 3.1 It's not easy, but it's worth it

The FLMs described their work situation as multifaceted and complex, and as affecting them in many ways. They described their job as meaningful; they truly enjoyed working as managers in elderly care. The responsibility they had been given was complex and demanding; it was combined with great authority within set boundaries, which helped them manage their great responsibility. Support from other persons in the organisation was essential to not feeling lonely in their role. They also reported getting support from organisational preconditions and confidence in their own abilities. However, when organisational preconditions were insufficient, the FLMs reported striving to deal with the situation. Table 3 presents an overview of the theme and subthemes.

**Table 3:** Overview of the theme and the four subthemes

It's not easy, but it's worth it	Enjoying a meaningful job
	A complex and demanding responsibility that allows great authority within set boundaries
	Supported by other persons, organisational preconditions and confidence in their own abilities
	Lacking organisational preconditions, but developing strategies for dealing with the situations

### 3.2 Enjoying a meaningful job

Many of the FLMs mentioned being dedicated to working with people, especially the elderly. The FLMs described that one personal goal was to be able to work closely with staff to provide the best possible care for their elderly clients. This goal also corresponded with the organisational goals. All FLMs enjoyed their work in elderly care, and many of them reported being proud of it. Being able to have an impact on the work and provide good elderly care together with staff were important to them. “I'm only this happy when I get to tell about the fantastic work we do here.” (Informant 7). To experience meaning in and enjoy their work, the FLMs needed their own goals and values to correspond with those of the organisation. Most of the FLMs described opportunities to advance within the organisation, but they had chosen to stay in their current positions to stay close to the day‐to‐day work. They enjoyed the long‐term interaction with the clients, relatives and staff. “I don't want another position, at heart, I've been offered other positions many times. But I like working as a first‐line manager,// well working directly with operations, that gives me energy.// having lots of balls in the air and being in an organization where I get direct feedback.” (Informant 9). Many of the FLMs described another meaningful and enjoyable part of their work, which was being able to use their leadership skills to make their staff feel seen and help them grow. The fact that their days were never the same and that the work tasks were varied, and challenging was very inspiring to the FLMs.

### 3.3 A complex and demanding responsibility that allows great authority within set boundaries

The FLMs described their responsibilities as complex and to some extent unclear, as for many, these responsibilities were not actually documented anywhere. Some FLMs said their great responsibilities were frightening, but also offered a positive challenge and were in this respect an attractive part of their work. Their responsibilities were broad and included staff, work environment, facilities, daily working processes, finances, clients and quality of care. Some of the FLMs described the different parts of their responsibilities as links in a chain. All parts were important and connected to each other, allowing them to provide the clients with high‐quality care. “Because I'm responsible for the staff I also have to manage the budget, or vice versa, because they go together. And because I'm responsible for the budget I also have responsibility for the organization itself. Because my revenues.// I usually think about it like a three‐legged table somehow. All three legs are equally important to making it work.” (Informant 11). However, although the FLMs described complex and demanding responsibilities, many of them also reported that as long as they fulfilled their responsibilities and worked within established boundaries, their authorities were extensive. This was positive, because upper management trusted them to make their own decisions. “The “hows” are all the things we can influence. The “whats” are the things we can't always influence.” (Informant 9). Some of the FLMs, however, expressed wanting to firmly establish decisions about major investments with their managers. Furthermore, most of the FLMs reported having the authority to make both long‐term and short‐term plans for their work, as long as they attended planned meetings and met deadlines; this gave them a sense of having control over their own work.

Having responsibility for subordinates was complex, as the FLMs were responsible for many different staff categories and the number of subordinates was often high. “… assistant nurses, there are nurses, occupational therapists and physical therapists, aid equipment technicians, night staff.. it's so broad// I mean 45, like I said, 45 assistant nurses wouldn't have been a problem, I don't think. But just the fact that they're spread across different work categories, so to speak// that's difficult.” (Informant 3). Another complex part of the FLMs' staff responsibilities was that many of them were responsible for several units, which in some cases were spread out over a large geographical area. Some of the FLMs' offices were not located in the same facility their staff worked in. Most of the FLMs prioritized being available to their staff around the clock and building team spirit; they did this so that they and their staff could jointly create good quality care for their clients. Although the informants enjoyed their staff responsibilities, they sometimes found them difficult to deal with, especially when staff did not conduct themselves well and failed to perform their duties. To give staff the best prerequisites to perform, the FLMs had to create a good work environment. However, the FLMs said that creating a good work environment was difficult, especially when staff worked in the clients' own homes, environments the FLMs had limited impact on. Another challenge for the FLMs was being responsible for the budget. The financial resources were limited, but the clients' needs varied considerably over time (and therefore also the need for staff), making it difficult to keep within the budget.

### 3.4 Supported by other persons, organisational preconditions and confidence in their own abilities

The results reveal that all FLMs experienced some form of support in their managerial work. For example, the coordinators offered hands‐on support with operative, administrative tasks, which helped them manage their heavy workload. “… if I didn't have a good coordinator I think that would mean a whole lot of work, really// it would be too much” (Informant 7). Colleagues offered them both hands‐on support with the managerial work and support in the form of advice or discussing matters. The support their own managers and the political board gave was described in different ways. Some FLMs reported having close relations with their managers, with daily encounters or phone calls and hands‐on support. Those FLMs said their managers understood the work situation of managers in the first‐line and they stood up for the FLMs in contacts with upper management and politicians. Other FLMs described a more distanced relation with their managers; they had to seek support from management by themselves, as no one reached out to them to offer support. Relations with politicians were described in similar ways. Some FLMs had close relations with politicians and could discuss their unit directly with them, whereas other FLMs described having to report to their managers, who in turn contacted the politicians.

Subordinates constituted another supportive resource, as the FLMs could delegate tasks to subordinates and subordinates supported the FLMs in their role. All of the FLMs mentioned receiving support from economists and payroll specialists, human resources specialists, computer management specialists and many others in the organisation. They also described different administrative systems that helped them with their administrative tasks. Support of all kinds was important to the informants in managing their great responsibility and high workload; it also made them feel less alone in their role. However, it was more common for the FLMs to have to seek support themselves than for someone within the organisation to actively offer it. Access to adequate information about the work was another supportive prerequisite that many organisations offered the informants. Support was also described as organisational preconditions, such as having access to supplementary training and working in different groups, where the FLMs could make an impact and get a broader understanding of the organisational context. Feeling confidence in their own abilities as FLMs created feelings of control and self‐confidence when dealing with daily difficulties in their work. Such feelings were considered necessary for them to handle the complex work. Many informants felt they did not have the right competence when beginning their work as FLMs, but they had “learned by doing,” which gave self‐esteem in their role and made them feel competent. “…at first I didn't think I had the right skills// but now that I've been doing this for almost 10 years// so much has happened during these years I feel pretty secure in my role really// that I do a good job.” (Informant 7).

### 3.5 Lacking organisational preconditions, but developing strategies for dealing with the situations

Although the previous subtheme dealt with support, the FLMs also reported times when their organisational preconditions gave insufficient resources in the form of time and finances, authority, information and support from others. This subtheme also concerns the FLMs' efforts to take back control and to deal with demanding situations. For example, the FLMs said that time was a scarce resource. The constant high workload and work involving “putting out fires” kept them from working with a cycle of follow‐up and long‐term planning on their units. “I wish I had more time, to plan and evaluate something I've done or something else, but things just keep rolling along. I … have gotten used to it, that's one way to manage too. To feel that this is just the way things are.” (Informant 1). Most of the informants also reported that increased administrative tasks and ineffectual meetings prevented them from focusing on core activities and being available to staff and clients. This was difficult for them, as they knew being available was important to keeping up to date on workplace events and preventing small problems from getting bigger. “… doing everything twice.// that's the difficulty of managing two places.// it would definitely have been better to be in one building. Being available all the time, because today not being on site is a problem. Because you notice things earlier when you're there.” (Informant 2). In order to take control and deal with these difficulties, the FLMs had learned how to plan and prioritize different work tasks. They also described strategies such as making themselves available to staff and clients by leaving the office door open and prioritizing daily encounters with staff. Another strategy described was being prepared to sacrifice one's own spare time to maintain control, as it was not possible to limit work to working hours only.

Some of the FLMs reported having reduced the number of staff to a minimum and not being able to cut back on staff any further to balance the budget. High number of subordinates and responsibility for more than one unit, combined with limited authority in hiring staff and making staff decisions, created feelings of frustration, stress and dissatisfaction with work. To take control of and deal with these circumstances, they had to be creative and innovative when scheduling staff, and sometimes they had to make decisions that were beyond their mandate. Furthermore, the FLMs sometimes experienced insufficiencies in information and support from management, politicians and supportive systems. They wished the organisations would offer them more hands‐on support with administrative tasks from coordinators, economists, the payroll department, the human resources department and the computer management department. Not all of the FLMs had someone who could replace them and assume responsibility for the units after hours, on weekends and when they were on vacation or sick leave, but all of the FLMs wished they had access to such a person. In order to manage their lonely role when support was lacking, some FLMs had built up informal networks with peers and staff who were supportive and made them feel secure. Because it takes a long time to build up informal networks, the FLMs who were new to the organisations did not have these kinds of supportive alliances.

Furthermore, some of the FLMs reported that politicians sometimes set political goals that were unattainable given their limited resources. Then, the informants had to be creative and innovative in their efforts to achieve these goals. “… I have told// the healthcare board's chair, politicians and my boss at XX that I have no intention of lower the staffing density goals, because that reduces quality for clients and then I won't stay on as first‐line manager// Instead I feel we offer good quality care as things are now, but more cutbacks would affect residents too much.// But we stay within the budget, but then I think “why the hell do we do that?” *laughs* Do too well with this. At some point I'd like to show that this doesn't work, but unfortunately it does.// you have to be inventive.// If you're creative, well… that's what it's all about.” (Informant 9). However, the FLMs described a dilemma in this regard: they were creative and innovative in their efforts to achieve political goals with limited resources, but they experienced that politicians did not understand the severity of the situation when it did not affect the budget. Finally, most of the FLMs said they wanted more freedom to make decisions about daily care provision on their units. In their view, various control systems in the organisation and lack of resources concerning time, staff and finances have prevented them from making such decisions.

## 4 DISCUSSION

The present study reveals that the FLMs perceived their work situation as “not easy, but worth it.” They described their work as demanding and entailing complex responsibilities, but also great authority. When the organisational preconditions were deficient, they had to use different strategies to feel empowered in their daily work and in relation to others in the organisation. However, the FLMs enjoyed their work and described it as meaningful; they reported receiving support in their role from other persons, organisational preconditions and having confidence in their own abilities.

Kanter's theory of structural empowerment emphasizes staff having good access to opportunities, information, resources and support. The FLMs described having good access to *opportunities* in the form of supplementary training, working in different groups and changing workplaces in the organisation. However, the FLMs expressed no desire to change workplaces because they enjoyed their current positions as FLMs. This is contradictory to what managers in elderly care have reported in earlier research. A previous review suggested that both career and educational pathways were non‐existent (Dwyer, 2011). Most FLMs reported having sufficient *information* to perform well in their work, but some experienced insufficient information from management, politicians and supportive systems, which is consistent with findings from other studies (Trus, Doran, Martinkenas, Asikainen, & Suominen, 2018). The FLMs reported lacking *resources* in the form of time, staff and finances, which is also in line with previous research (Hagerman et al., 2015; Kristiansen et al., 2016; Udod, Cummings, Care, & Jenkins, 2017). Lack of resources causes stress among FLMs, as it is associated with lower performance and job satisfaction (Håkanson et al., 2014). Having a large number of subordinates, responsibility for different staff categories, for more than one unit and for units at different sites were challenges that prevented the FLMs from being present and available to their subordinates. According to previous research by Wong et al. (2015), having a large span of control positively predicts role overload and negatively predicts work control and job satisfaction among managers. In order to take control, the FLMs described how they had to be creative and innovative when trying to achieve organisational goals with limited resources. The FLMs often mentioned receiving *support*, but organisational preconditions sometimes failed to provide support, which could result in feelings of insufficiency, leading to stress and feelings of powerlessness. This is a serious situation for the FLMs, as stress has been reported to negatively affect leadership behaviours, decision‐making and to increase the risk of errors (Shirey, Ebright, & Mcdaniel, 2013). Lack of support has been reported to be related to managers' intention to leave their work (Skytt, Ljunggren, & Carlsson, 2007). In order to take back control and deal with situations of inadequate organisational support, the FLMs had built up *informal networks* of peers and staff (c.f. Kanter's description of informal power) who were supportive and made them feel secure (cf. Håkanson et al., 2014).

Regarding psychological empowerment (i.e., meaning, competence, self‐determination and impact), the FLMs enjoyed working in elderly care, found it *meaningful* and were proud of their work. They described the advantage of working closely with staff to give clients the best care possible. The importance of being able to work with their leadership and being a team builder was also described in a study of male FLMs' working life (Hagerman et al., 2015) and highlighted in another study on FLMs in elderly care (Håkanson et al., 2014). When describing their own *competence*, the FLMs expressed trust in their own experience in the position, which was essential in dealing with the complex work. This result is positive, as a previous review (Dwyer, 2011) suggested that nurse managers experienced lack of confidence in their role because they had not received sufficient professional training. Many informants reported lacking the right competence initially, but they had “learned by doing,” creating a feeling of competence and self‐confidence. According to previous research, this feeling is positively related to outcomes of leadership programs based on structural and psychological empowerment (Macphee, Skelton‐Green, Bouthillette, & Suryaprakash, 2012). As for *self‐determination*, the FLMs described their authority as sufficient, as long as they met their responsibilities and worked within set boundaries. When describing their *impact* on long‐term and short‐term planning for their units, they often reported not having enough time to engage in planning activities. The FLMs' descriptions about the organisations' management system lead to a secondary analysis of a number of interviews from the present study and from the study of male FLMs (Hagerman et al.,^2015^). A deductive analysis with a business management perspective showed that FLMs struggled with the differences in management approaches they experienced between the local, their, and central management. These differences are sometimes natural but it is of importance that managers deal with and make the most of them as it might influence the FLMs self‐determination (Strömberg, Engström, Hagerman, & Skytt, 2019). Labrague et al., (2018) describes the importance for managers to feel they have self‐determination and impact, as this helps them to deal with stressful managerial work.

It is interesting that the female FLMs described their work situation in a positive manner, including strategies for dealing with difficult situations, given that, in the previous study (Hagerman et al., 2015), all male FLMs described their work situation in more negative terms. In that study, the male FLMs did not clearly describe strategies for taking control of or dealing with difficult situations, but instead expressed feelings of frustration, dejection and resignation when organisational preconditions were insufficient. Could it be that the male FLMs were tokens, that is belonged to a minority group (Kanter, 1993), and that this made them feel more pressure to perform and more vulnerable than their female FLMs counterparts, who belonged to the majority group? Keisu (2009) used Kanter's (1993) structures of proportions when studying female FLMs in a male‐dominated manufacturing industry and male FLMs in a female‐dominated elderly care organisation. She found that being female tokens in a male‐dominated manufacturing industry had primarily negative connotations, as the expectations and demands placed on them were higher than those placed on the men in the majority group. Meanwhile, no negative consequences of being male tokens in a female‐dominated organisation were described by the male FLMs. Finegan and Spence Laschinger's (2001) survey of male token nurses and their female colleagues, found no difference in access to structural empowerment. However, few qualitative studies have investigated working life among FLMs in elderly care, especially from a theoretical perspective. Therefore, more studies are needed if we are to gain a deeper understanding of FLM's work situation, where this understanding is grounded in a theoretical perspective.

## 5 CONCLUSIONS AND IMPLICATIONS FOR NURSING MANAGEMENT

Our results highlight how important structural empowerment is for FLMs' experience of psychological empowerment. Although the FLMs did not always have sufficient access to structural empowerment, they experienced psychological empowerment in the form of meaning, competence, self‐determination and impact in their work. Because having structural empowerment is important to FLMs, it is important that upper management understand how they can offer FLMs such empowerment. One approach is to provide FLMs with more resources in the form of hands‐on support with administrative tasks and to lower their number of subordinates. This may reduce the work strain, allowing FLMs to be more available to their subordinates. Furthermore, the FLMs' emphasized having access to a person who could replace them and assume responsibility for the units when the FLMs were on sick leave or vacation. Upper management needs to ensure that support is offered when needed and that each FLM is offered customized support based on her/his own needs.

## ETHICAL APPROVAL

Regional Ethical Review Board in Uppsala, Sweden (reg. no. 2010/192).
